# Area-Level Attributes and Esophageal Adenocarcinoma in Surveillance, Epidemiology and End Results Registries

**DOI:** 10.1371/journal.pone.0081613

**Published:** 2013-11-11

**Authors:** Armen A. Ghazarian, Megan A. Murphy, Maria R. Khan, Brit I. Saksvig, Sean F. Altekruse

**Affiliations:** 1 Division of Cancer Epidemiology and Genetics, National Cancer Institute, Bethesda, Maryland, United States of America; 2 Department of Epidemiology and Biostatistics, University of Maryland School of Public Health, College Park, Maryland, United States of America; 3 Division of Cancer Control and Population Sciences, National Cancer Institute, Bethesda, Maryland, United States of America; Kyushu University Faculty of Medical Science, Japan

## Abstract

**Purpose:**

To examine the associations between area-level socioeconomic attributes and stage of esophageal adenocarcinoma diagnoses in 16 SEER cancer registries during 2000-2007.

**Methods:**

Odds ratios (OR) and 95% confidence intervals (CI) were calculated using multivariable logistic regression models to assess the relationship between distant-stage esophageal adenocarcinoma and individual, census tract, and county-level attributes.

**Results:**

Among cases with data on birthplace, no significant association was seen between reported birth within versus outside the United States and distant-stage cancer (adjusted OR=1.02, 95% CI: 0.85-1.22). Living in an area with a higher percentage of residents born outside the United States than the national average was associated with distant-stage esophageal adenocarcinoma; census tract level: >11.8%, (OR=1.10, 95% CI:1.01–1.19), county level: >11.8%, (OR=1.14, 95% CI:1.05-1.24). No association was observed between median household income and distant-stage cancer at either census tract or county levels.

**Conclusion:**

The finding of greater odds of distant-stage esophageal adenocarcinoma among cases residing in SEER areas with higher proportion of non-U.S. Natives suggests local areas where esophageal cancer control efforts might be focused. Missing data at the individual level was a limitation of the present study. Furthermore, inconsistent associations with foreign birth at individual- versus area-levels cautions against using area-level attributes as proxies for case attributes.

## Background

In the United States, it is estimated that 14,440 men and 3,550 women will be diagnosed with and 15,210 people will die of cancer of the esophagus in 2013 [1]. The incidence rate for esophageal cancer significantly increased from 1975 through 2009 in Surveillance, Epidemiology and End Results (SEER 9) Registries [2]. Esophageal adenocarcinoma arises from glandular cells present at the junction of the esophagus and stomach [3]. There are two major histological types of the cancer: squamous cell carcinoma and adenocarcinoma. While squamous cell carcinoma incidence rates have decreased, rates for adenocarcinoma have increased over 6-fold in the United States since the 1970s [2]. Most of the increasing incidence and death rates are attributed to the rising burden of esophageal adenocarcinoma experienced among white males [4].

Because the esophagus is located near other vital organs, lesions are most treatable when detected as the premalignant condition of Barrett’s esophagus [5] or as early-stage cancer. In SEER Registries, overall 5-year esophageal cancer survival increased from 5 to 20% from the 1970s through 2004. Furthermore, from 2001 to 2007, 5-year esophageal cancer survival was 38% among localized-stage cases and 20% among regional-stage cases, but only 3% among distant-stage cases [2]. Although there is currently no standard or routine screening test for esophageal cancer, detection of Barrett’s esophagus and early-stage esophageal cancers are still possible through medical procedures including endoscopy, biopsy, brush and balloon cytology, chromoendoscopy, and fluorescence spectroscopy [6]. Further experimental studies are needed to evaluate the effectiveness of these screening procedures for esophageal cancers.

Low socioeconomic status may be a risk factor for esophageal adenocarcinoma [7,8]. Specific socioeconomic factors that are significantly associated with increased EAC risk include education [8], wealth [8], occupational status [7], and marital status [7,8]. Other established risk factors for esophageal adenocarcinoma include older age [5,9,10], male gender [9,10], white race [11], obesity [9], and smoking [5,9,10,12,13].

Social support may encourage lifestyle and screening behaviors that reduce the risk of esophageal adenocarcinoma [7]. Positive social support can confer health benefits in several ways [14], including improving awareness of and access to available health care resources and reducing behavioral risk factors that predispose to esophageal adenocarcinoma. In SEER Registries, we examined associations between esophageal adenocarcinoma stage, case characteristics, and area-level attributes associated with this diagnosis including median household income and percent foreign-born population. 

## Methods

The NCI IRB and the IRB of the contributing SEER registries have granted a waiver from HIPAA requirements because the SEER data used in this analysis have been de-identified, protecting against disclosure of personal information. The study was restricted to incident cases from 16 Surveillance, Epidemiology and End Results Registries that were diagnosed between the years 2000 and 2007. The Registries included eight states (New Mexico, Hawaii, Utah, Iowa, Connecticut, Kentucky, Louisiana, New Jersey) and eight areas (Atlanta, Rural Georgia, Greater California, San Francisco-Oakland, San Jose/Monterey, Los Angeles, Seattle, Detroit), which together represent approximately 20 percent of the United States [15]. Census tract- and county-level census data regarding the percent foreign-born population were obtained from the Census Bureau [16].

### Case Identification

Cancer site and histology were defined by the International Classification of Diseases for Oncology (ICD-O) (3rd edition) codes: esophagus (C15.0-C15.9) and adenocarcinoma (8140-8573). These criteria identified 14,264 esophageal adenocarcinoma cases in SEER registries during the surveillance period.

Of 14,264 reported esophageal adenocarcinoma cases, 11,233 had a single primary tumor. Only cases with a single primary tumor were included in this study. Of these, 1,134 cases were excluded due to missing stage. The resulting analytic dataset was comprised of 10,099 cases with localized-, regional-, or distant-stage esophageal adenocarcinoma at initial diagnosis; 71% of all esophageal adenocarcinoma cases in the SEER dataset.

### Case and Area-Level Attributes

The analysis linked esophageal adenocarcinoma cases to census tract- and county-level data regarding the percent of the population born outside the United States. Areas were stratified into groups with less than or equal to 11.8% foreign-born population: the estimated national mean percent foreign-born population in 2002 [17] with sensitivity analyses based on quartiles of foreign born population. In addition, four individual-level variables were included in the analysis: age, race, gender, and place of birth. Data for most of these demographic variables were primarily abstracted from medical records. Death certificates were, however, an important source of place of birth. The outcome variable was SEER historic stage at diagnosis (i.e., localized and regional versus distant stage).

### Statistical Analysis

The overall distribution of esophageal adenocarcinoma cases by individual and area-level attributes was explored, as well as by stage at diagnosis. Logistic regression models were developed to estimate odds ratios and 95% confidence intervals for associations between individual and area-level attributes and distant-stage esophageal adenocarcinoma diagnosis. Crude and adjusted models accounted for age group (≤ 69, 70+), year of diagnosis (2000-2007), sex, and race (white, other). Statistical analyses were conducted using SAS (SAS v. 9.2., Cary, NC).

## Results

### Demographic and Registry Distributions

Individual case-level demographic data for esophageal adenocarcinoma cases are presented by stage at diagnosis (Table 1). The peak age groups of diagnosis were 50-69 years of age (52%), followed by 70-84 years of age (33%). Approximately 90% of cases were non-Hispanic whites. At all stages at diagnosis, the vast majority of cases (> 85%) were male. Although nearly one-third of cases were missing data on place of birth, at least 64% of cases in this report were born in the United States.

**Table 1:** Individual and area-level attributes by stage at diagnosis, esophageal adenocarcinoma incident cases, SEER Registries, 2000-2007.

	All Cases, n= 10099	Localized/Regional, n= 6114	Distant, n= 3985
**Individual Level**			
Age at Diagnosis (n, %)			
20-49 years	1021 (10.1)	514 (8.4)	507 (12.7)
50-69 years	5295 (52.4)	3077 (50.3)	2218 (55.7)
70-84 years	3293 (32.6)	2171 (35.5)	1122 (28.1)
≥ 85 years	490 (4.9)	352 (5.8)	138 (3.5)
Race/Ethnicity (*n*, %)			
White	9036 (89.5)	5560 (90.9)	3476 (87.2)
Hispanic (All Races)	579 (5.7)	296 (4.8)	283 (7.1)
Black	231 (2.3)	123 (2.0)	108 (2.7)
Asian/Pacific Islander	188 (1.9)	100 (1.6)	88 (2.2)
American Indian/Alaska Native	34 (0.3)	15 (0.3)	19 (0.5)
Other/Unknown	31 (0.3)	20 (0.3)	11 (0.3)
Sex (*n*, %)			
Male	8688 (86.0)	5278 (86.3)	3410 (85.6)
Female	1411 (14.0)	836 (13.7)	575 (14.4)
Place of Birth (*n*, %)			
US	6486 (64.2)	3692 (60.4)	2794 (70.1)
Foreign	567 (5.6)	318 (5.2)	249 (6.3)
Unknown	3046(30.2)	2104 (34.4)	942 (23.6)
**Census Tract Level**			
Percent Foreign Born (n, %)			
> 11.8%	3887 (38.5)	2295 (37.5)	1592 (40.0)
≤ 11.8%	6212 (61.5)	3819 (62.5)	2393 (60.0)
Median Household Income (*n*, %)			
> 54.0K	3663 (36.3)	2195 (35.9)	1468 (36.8)
> 45.6 to 54.0K	1768 (17.5)	1083 (17.7)	685 (17.2)
40.8 to 45.6K	1066 (10.5)	648 (10.6)	418 (10.5)
≤ 40.8K	3602 (35.7)	2188 (35.8)	1414 (35.5)
**County Level**			
Percent Foreign Born (n, %)			
> 11.8%	4703 (46.6)	2769 (45.3)	1934 (48.5)
≤ 11.8%	5396 (53.4)	3345 (54.7)	2051 (51.5)
Median Household Income (*n*, %)			
> 54.0K	3960 (39.2)	2388 (39.1)	1572 (39.4)
> 45.6 to 54.0K	2698 (26.7)	1636 (26.8)	1062 (26.7)
40.8 to 45.6K	1657 (16.4)	981 (16.0)	676 (17.0)
≤ 40.8K	1784 (17.7)	1109 (18.1)	675 (16.9)

### Census Tract and County-Level Attributes

At both the census tract and county level, the majority of cases lived in areas with equal or lower percent foreign-born population than the U.S. average in 2002, 11.8% (Table 1). Nearly 35% of cases lived in census tracts with a median household income less than or equal to $40,800, and 18% of cases lived in counties with median household incomes of less than or equal to $40,800. These distributions were relatively consistent across all stages of diagnosis. 

### Association with Stage at Diagnosis

Cases 70 years of age and older were less likely than younger cases to be diagnosed with distant-stage esophageal adenocarcinoma (adjusted odds ratio (OR)=0.65, 95% confidence interval (95% CI) from 0.60 to 0.71), (Table 2). In addition, white cases were less likely to be diagnosed with distant-stage cancer than cases of other racial origin (OR=0.76, 95% CI from 0.63 to 0.91). When male cases were compared to female cases, males had lower odds of being diagnosed with distant-stage cancer (OR=0.88, 95% CI from 0.79 to 0.99). Further stratification by year of diagnosis revealed the association to be attenuated in all but one year (2003) with no evidence of a time trend that could be explained by changes in screening practices (data not shown). After excluding cases with missing data, no association was seen between reported birth in the United States versus non-U.S. place of birth and distant-stage cancer (OR=1.02, 95% CI from 0.85 to 1.22). The null association between nativity and stage persisted in analyses restricted to registries with the most complete data on place of birth, including three registries with 88% completeness: Hawaii, Connecticut, and Metropolitan Atlanta (data not shown). Of note, place of birth was more likely to be missing for cases with localized/regional stage of disease, younger than 70 years of age, and diagnosed in more recent years. The same findings were evident in subanalyses restricted to the three registries with the most complete data on place of birth (data not shown).

**Table 2:** Associations between stage at diagnosis and case attributes, esophageal adenocarcinoma incident cases, SEER Registries, 2000-2007.

	n	Crude OR	(95% CI)	Adjusted OR†	(95% CI)
**Individual Level**					
Age at Diagnosis					
≥ 70 years	3783	0.66	(0.61-0.72)	0.65	(0.60-0.71)
≤ 69 years	7216	1.00	Referent	1.00	Referent
Race/Ethnicity					
White	9610	0.73	(0.61-0.87)	0.76	(0.63-0.91)
Other*	489	1.00	Referent	1.00	Referent
Sex					
Male	8688	0.94	(0.84-1.05)	0.88	(0.79-0.99)
Female	1411	1.00	Referent	1.00	Referent
Place of Birth					
Foreign	567	1.04	(0.87-1.23)	1.02	(0.85-1.22)
US	6486	1.00	Referent	1.00	Referent
**Census Tract Level**					
Percent Foreign Born					
> 11.8%	3887	1.11	(1.02-1.20)	1.10	(1.01-1.19)
≤ 11.8%	6212	1.00	Referent	1.00	Referent
Median Household Income					
> 54.0K	3663	1.04	(0.94-1.14)	1.04	(0.95-1.15)
> 45.6 to 54.0K	1768	0.98	(0.87-1.10)	1.00	(0.89-1.12)
40.8 to 45.6K	1066	1.00	(0.87-1.15)	1.01	(0.87-1.16)
≤ 40.8K	3602	1.00	Referent	1.00	Referent
*test for trend*			*P= 0.53*		*P= 0.40*
**County Level**					
Percent Foreign Born					
> 11.8%	4703	1.14	(1.05-1.23)	1.14	(1.05-1.24)
≤ 11.8%	5396	1.00	Referent	1.00	Referent
Median Household Income					
> 54.0K	3960	1.08	(0.96-1.20)	1.08	(0.96-1.22)
> 45.6 to 54.0K	2698	1.07	(0.94-1.21)	1.06	(0.94-1.20)
40.8 to 45.6K	1657	1.13	(0.99-1.30)	1.14	(0.99-1.30)
≤ 40.8K	1784	1.00	Referent	1.00	Referent
*test for trend*			*P= 0.41*		*P= 0.39*

^†^ Adjusted for age, sex, race, and year of diagnosis

* Includes all races and ethnicities combined excluding whites

At the census tract level, cases living in areas with a greater proportion of foreign-born population than the U.S. average (11.8%) had higher odds of distant versus localized-stage esophageal adenocarcinoma compared with cases living in census tracts with a lower proportion of foreign-born residents (adjusted odds ratio (OR)=1.10, 95% CI from 1.01 to 1.19). The association was statistically significant for cases residing in the highest quartile areas of foreign-born population (data not shown). Median household income was not associated with stage at diagnosis at the census tract level (p-trend crude = 0.53; p-trend adjusted = 0.40). 

At the county level, cases living in areas with greater than 11.8% of the population born outside the United States also had statistically significant higher odds ratios of distant-stage esophageal adenocarcinoma compared to cases living in counties with a lower proportion of foreign-born residents (adjusted OR=1.14, 95% CI from 1.05 to 1.24). The association was statistically significant for the highest two quartiles of area foreign-born population (data not shown). Median household income was not associated with stage at diagnosis at the county level (p-trend crude = 0.41; p-trend adjusted = 0.39). 

The association between distant-stage EAC and socioeconomic attributes persisted when compared separately with localized then regional stage cases (data not shown), however, the effect was most clearly illustrated in the final model (localized/regional versus distant). In a subanalysis including cases with multiple primary tumors, crude and adjusted models for all socioeconomic attributes remained essentially unchanged (data not shown). Finally, in analyses restricted to cases born outside the United States, area-level associations with foreign born populations were no longer statistically significant (data not shown).

## Discussion

In this study, no association was seen between reported birth inside the United States versus outside the country and distant-stage esophageal cancer. While the majority of cases were born in the United States, at both the census tract and county level, cases that lived in areas with a high percentage of non-U.S. born residents had significantly higher odds of distant-stage esophageal adenocarcinoma. This finding has potential implications for cancer control because screening to detect early-stage cancer may improve prognosis [6]. The findings also demonstrate that area-level and individual attributes can operate independently, with the potential for misclassification if area-level measures are used as proxies for personal attributes. 

At the individual level, the risk of developing esophageal adenocarcinoma is associated with limited social support and low socioeconomic status [7,8]. We were, however, unable to identify studies that related esophageal adenocarcinoma stage at diagnosis to area-level percent foreign-born population. Studies of other cancer sites may be informative in this regard. In SEER registries, Hispanics living in neighborhoods with higher density Hispanic populations were more likely to be diagnosed with late-stage breast, cervical, and colorectal cancers [18]. Foreign-born Hispanic women in California residing in high-enclave neighborhoods were also more likely than U.S.- born Hispanic women who reside in assimilated neighborhoods to be diagnosed with distant-stage breast cancer [19]. Low income, poor health insurance coverage, and language barriers are also often experienced within less assimilated Hispanic communities [20]. Another interesting finding at the individual level was the lower risk of distant-stage EAC for men compared to women. This association was further strengthened in the adjusted model. Men have a higher risk of developing EAC and Barrett’s esophagus, therefore, they may be identified earlier through screening. Since screening practices for EAC have changed over time with no clear guidelines for a standard screening procedure, we further stratified by year of diagnosis, however, no clear time trend was evident that could be explained by screening. 

Keegan and colleagues hypothesized that the surplus of distant-stage cancer in enclave communities was a function of limited access to screening [19]. Low income [21], poor health insurance coverage [22], and language barriers [18] are often encountered in incompletely assimilated communities [20]. Thus, low social position in areas with sizeable non-native populations may contribute to the association in the present report between high area-level percent foreign-born population and distant-stage esophageal adenocarcinoma. Better access to health care [22] and higher SES [18] may contribute to the finding that white cases in our study were less likely to be diagnosed with distant-stage esophageal adenocarcinoma than non-white cases. 

Strengths of the study included the large sample size and linkage to area-level attributes. There was, however, limited availability of individual-level socioeconomic data, and nearly 30% of cases were missing data regarding place of birth. A large proportion of cases with missing place of birth had localized/regional stage of disease, were younger than 70 years of age, and were diagnosed in more recent years. We suspect that place of birth is most complete among cases with worse outcomes because death certificates are a common source of this information. Nonetheless, stratified analyses in registries with the most complete data on nativity supported the null finding. A limited amount of demographic data is typically collected in cancer registries. To overcome this limitation of cancer surveillance, area-level data are sometimes used as proxies for individual data. This practice is based on the assumption that people residing in a common area often share similar socioeconomic attributes. As our findings suggest, caution is warranted against this practice, because area-level measures can operate differently from individual-level measures. First, attributes of residents can differ from prevailing attributes. In addition, while area-level socioeconomic status affects social support and quality of care within a community [20], it occurs at the ecologic level. 

In summary, while residing in an area with a high percent of non-U.S. born individuals was associated with distant-stage esophageal adenocarcinoma, no such association was evident among cases with individual level data on place of birth. The findings suggest a need for caution regarding the use of area-level measures as proxies for individual level attributes. The findings of this study could also help target resources toward specific neighborhoods and individuals at risk for late-stage esophageal adenocarcinoma. 
